# Abundances of Clinically Relevant Antibiotic Resistance Genes and Bacterial Community Diversity in the Weihe River, China

**DOI:** 10.3390/ijerph15040708

**Published:** 2018-04-10

**Authors:** Xiaojuan Wang, Jie Gu, Hua Gao, Xun Qian, Haichao Li

**Affiliations:** College of Natural Resources and Environment, Agriculture Key Laboratory of Plant Nutrition and Agri-Environment in Northwest China, Northwest A&F University, Yangling 712100, China; xiaojuan7069@sina.com (X.W.); gaohua1112@163.com (H.G.); qianxun@nwsuaf.edu.cn (X.Q.); lihaichaojy@163.com (H.L.)

**Keywords:** antibiotic resistance gene, bacterial community, droplet digital polymerase chain reaction (ddPCR), network analysis, Weihe River

## Abstract

The spread of antibiotic resistance genes in river systems is an emerging environmental issue due to their potential threat to aquatic ecosystems and public health. In this study, we used droplet digital polymerase chain reaction (ddPCR) to evaluate pollution with clinically relevant antibiotic resistance genes (ARGs) at 13 monitoring sites along the main stream of the Weihe River in China. Six clinically relevant ARGs and a class I integron-integrase (*intI1*) gene were analyzed using ddPCR, and the bacterial community was evaluated based on the bacterial 16S rRNA V3–V4 regions using MiSeq sequencing. The results indicated Proteobacteria, Actinobacteria, Cyanobacteria, and Bacteroidetes as the dominant phyla in the water samples from the Weihe River. Higher abundances of *bla*_TEM_, *strB*, *aadA*, and *intI1* genes (10^3^ to 10^5^ copies/mL) were detected in the surface water samples compared with the relatively low abundances of *strA*, *mecA*, and *vanA* genes (0–1.94 copies/mL). Eight bacterial genera were identified as possible hosts of the *intI1* gene and three ARGs (*strA*, *strB*, and *aadA*) based on network analysis. The results suggested that the bacterial community structure and horizontal gene transfer were associated with the variations in ARGs.

## 1. Introduction

In recent years, antibiotic resistance genes (ARGs) have been recognized as emerging environmental contaminants due to the overuse and misuse of antibiotics in medical, veterinary and agricultural applications [1,2]. ARGs can be widely diffused among different bacterial species in the environment through horizontal gene transfer (HGT) [3,4,5] and ARGs circulating in the environment can significantly affect microbial population dynamics, and pose potential threats to public health through the food chain [6,7]. In addition, HGT may facilitate the transfer of ARGs from non-pathogens to human pathogens when both coexist in the environment, thereby causing antibiotic resistance and the subsequent failure of infectious disease treatments [6,8]. Most ARGs are located on mobile genetic elements such as plasmids and transposons [5,9]. Integrons are involved with the transfer of ARGs between different bacterial species due to their association with plasmids [10]. Therefore, integrons play an important role in the dynamics of antibiotic resistance. Among the three major integrons (classes 1, 2, and 3) [10], the class I integron-integrase (*intI1*) genes are the most common gene capture and transmission system in clinical and environmental isolates [11]. Recently, the *intI1* gene was selected as a generic marker for evaluating anthropogenic pollutants because of its common association with antibiotic resistance in both pathogenic and non-pathogenic bacteria [3,12].

Streams and rivers are important for human culture, welfare, and development, but they are also a key route for the dissemination of ARGs [13]. Various antibiotics are used extensively for clinical treatments or on farms in China, which may induce antibiotic resistance in bacterial pathogens [2]. Therefore, the surveillance of ARGs in surface rivers is critically important for ensuring water quality and public health [14]. Among the ARGs identified in previous studies, those related to sulfonamide and tetracycline resistance are the most common in Chinese aquatic environments, followed by β-lactam ARGs [11,14,15,16]. 

Studies have detected ARGs in surface river systems but most of these previous studies focused on cultivable bacteria [17,18]. Current molecular approaches such as the polymerase chain reaction (PCR) and real-time quantitative PCR (qPCR) are faster, and they can alleviate the main problems related to culture-based methods, which are used most often for detecting ARGs in aquatic environments [19,20,21]. However, standardization based on reference materials limits the application of qPCR for detecting ARGs in environmental samples [22]. Furthermore, qPCR is susceptible to inhibition by common constituents found in environmental samples, such as humic and fulvic acids, which are major inhibitory substances [23,24]. Recently, droplet digital PCR (ddPCR) has been developed based on partitioning (to mimic limiting dilution) and Poisson statistics, which overcomes the potential limitations of qPCR [25]. The major advantages of ddPCR compared with conventional qPCR include the simple experimental procedure employed and the high sensitivity at detecting trace DNA in samples. Dilution curves and PCR efficiency calculations for DNA standards are not required for absolute quantification [26]. In addition, ddPCR is more accurate than qPCR for nucleic acid detection because it allows absolute quantification of nucleic acids in samples without the need for external calibrators. Recently, ddPCR has been successfully applied in DNA assays using environmental samples, such as for ARGs in soils and organic residues [23], fecal indicators in water [22], and *Escherichia coli* in bovine feces [27].

**Figure 1:**
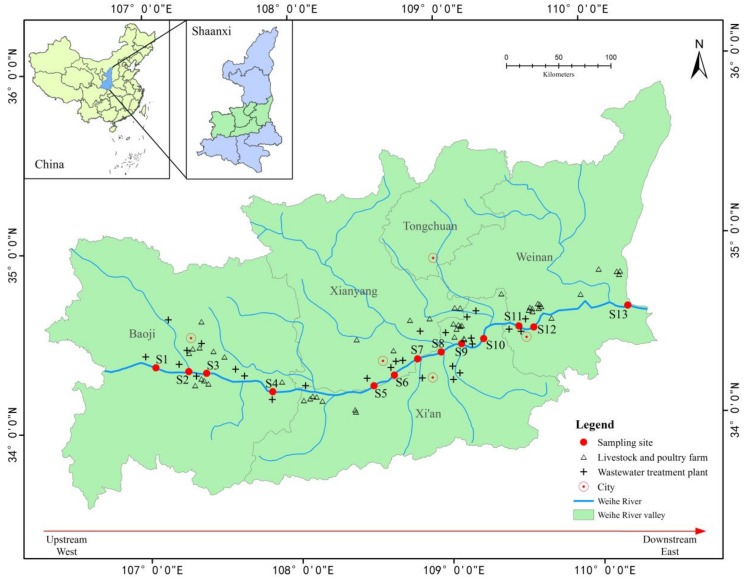
Locations of the sampling sites.

The Yellow River is the second longest river in China and it has experienced severe loading with pollutants during recent decades [28]. The Weihe River is the largest tributary of the Yellow River and it is located in north-west China (Figure 1). The Weihe River is known as the “Mother River” of the Guanzhong region and it plays important roles in the social, ecological, and economic development of Shaanxi Province. In Shaanxi Province, about 64% of the population, 56% of the farmland, 72% of the irrigation area, and 82% of the total industrial output value are found in the Weihe River area [29]. This area receives large amounts of sewage effluent and animal waste discharges, which may contain various antibiotics and antibiotic-resistant bacteria. According to survey data from the Shaanxi Provincial Environmental Protection Bureau, 245 sewage discharge points are distributed on both sides of the Weihe River and more than 700 million tons of sewage are discharged into the river annually [30]. Thus, contamination by ARGs, especially those that are clinically relevant, is a severe challenge that affects water quality and public health in the Weihe River area. The distributions of clinically relevant ARGs in the Weihe River and the diversity of the bacterial community have not been evaluated previously.

In this study, we evaluated clinically relevant ARGs in the Weihe River using ddPCR, i.e., two β-lactam resistance genes (*bla*_TEM_ and *mecA*), one vancomycin resistance gene (v*anA*), three streptomycin resistance genes (*strA*, *strB*, and *aadA*), one integron-integrase gene (*intI1*), and the 16S rRNA gene. The bacterial community was analyzed based on the 16S rDNA V3–V4 regions using MiSeq sequencing. The aims of this study were to evaluate the levels of pollution by clinically relevant ARGs and the spatial variation in the bacterial community in the Weihe River. The results of this study may allow the identification of potential factors related to the spatial distribution of ARGs in the Weihe River as well as clarifying the relationships between ARGs, bacterial communities, and environmental factors. To the best of our knowledge, this is the first study to investigate clinically relevant ARGs in water samples using ddPCR. 

## 2. Materials and Methods 

### 2.1. Sampling Sites and Sample Collection

Water samples were collected from 13 monitoring stations along the main stream of the Weihe River during November 2015 (Supplementary Materials, Table S1). Figure 1 shows the 13 monitoring sites along the main stream of the Weihe River in Shaanxi province. Four cities are located along the main stream from west to east, i.e., Baoji, Xianyang, Xi’an and Weinan. Xi’an City is the capital of Shaanxi Province and it had a total population of 8.5 million in 2010. Xi’an City is one of the cradles of ancient Chinese civilization in the Yellow River Basin and the starting point of the Silk Road [29]. Samples were collected along the Weihe River (Shaanxi section) in the western part (sampling sites S1–S4), middle part (S5–S10), and eastern part (S11–S13). Seven sites (S2–S3, and S8–S12) were located in urban areas and six sites (S1, S4–S7, and S13) were located in rural areas. The water samples were collected aseptically from 10–20 cm below the surface in sterile polyethylene bottles (1000 mL). The electrical conductivity, temperature, pH and dissolved oxygen content were measured onsite at each sampling point using a calibrated HACH HQ30d system (HACH, Loveland, CO, USA). Three bottles were sampled from each site and they were then returned to the laboratory in a portable icebox and processed within 24 h for further analyses. 

### 2.2. Water Sample Processing and Genomic DNA Extraction

Each water sample (1000 mL) was filtered through a porous mixed cellulose ester filter membrane (pore size = 0.22 μm) to collect cell pellets. The membrane filter was then cut into small pieces and mixed with 50% ethanol for fixation. All of the fixed samples were stored at −20 °C before subsequent DNA extraction. Total genomic DNA was extracted from the pieces of membrane using an E.Z.N.A. Water DNA kit (Omega, Biotech, Norcross, GA, USA) according to the manufacturer’s protocol. The concentration and purity of DNA were determined using an Epoch™ Multi-Volume Spectrophotometer System (Epoch, BioTek, Winooski, VT, USA) and the DNA samples were stored at −80 °C until their analysis.

### 2.3. Quantification of Antibiotic Resistance Genes (ARGs) Using Droplet Digital Polymerase Chain Reaction (ddPCR)

ddPCR was performed using a QX200™ Droplet Digital™ PCR system (Bio-Rad, Foster City, CA, USA). The reactions were conducted in a final volume of 20 μL containing 10 μL of 2× ddPCR Supermix for probes, 1 μL of primers and probes (final concentration of 900 nM for each primer and 250 nM for each probe), and 1 μL of the sample DNA. The primers and probes used in this study are shown in Supplementary Materials Table S2 [31,32,33,34,35]. The reaction mixtures were equilibrated to room temperature for 3 min before loading into a DG8^TM^ cartridge for droplet formation with a QX200 droplet generator (Bio-Rad, Foster City, CA, USA) according to the manufacturer’s instructions. The droplets obtained were transferred onto a 96-well PCR plate (Eppendorf AG, Hamburg, Germany) and heat-sealed at 180 °C for 5 s with a foil plate seal (Eppendorf AG, Hamburg, Germany). Thermal cycling was performed using a ProFlex^TM^ PCR system (Applied Biosystems, Carlsbad, CA, USA) with the following program: 95 °C for 5 min, 40 cycles at 94 °C for 30 s and 60 °C for 60 s, followed by 98 °C for 10 min, with a ramp rate of 2 °C/s. After PCR, the plate was transferred to a QX200 droplet reader (Bio-Rad, Foster City, CA, USA) for data acquisition. The fluorescence of each droplet was recorded in each well and automatically analyzed using QuantaSoft™ Software 1.7.4.0917.

### 2.4. Illumina High-Throughput Sequencing 

The extracted DNA samples were sent to the Beijing Center for Physical and Chemical Analysis (Beijing, China) for PCR amplification of the bacterial 16S rRNA gene and high-throughput sequencing. The 16S V3–V4 region was amplified using the primer pairs 341F (5′-CCTACGGGNGGCWGCAG-3′) and 805R (5′-GACTACHVGGGTATCTAATCC-3′) with different barcodes. All of the PCR products were then sequenced using the Illumina MiSeq platform (Illumina, San Diego, CA, USA). The raw reads were deposited in the National Center for Biotechnology Information (NCBI) Sequence Read Archive database (accession number SRP099541).

The sequences obtained from each sample were processed using the QIIME software package 1.6.0 [36]. After sequencing, the raw fastq files were demultiplexed and quality filtered. The reads were then merged in pairs into a new read based on their overlap using Fast Length Adjustment of SHort reads (FLASH, version 1.2.7, http://ccb.jhu.edu/software/FLASH/). The clean tags were checked and removed after testing for the presence of chimeras using UCHIME [37]. Next, the high-quality sequences were assigned to generate operational taxonomic units (OTUs) at 97% similarity with Usearch (version 7.1, http://drive5.com/uparse). Taxonomic assignments were then made for the sequences based on the Greengenes database using the RDP classifier (Version 2.2, http://sourceforge.net/projects/rdp-classifier/) [38]. The distributions of phyla in the 13 water samples were visualized using Circos (Version 0.67, http://circos.ca/). Subsequently, alpha and beta diversity analyses were both conducted with the mothur pipeline.

### 2.5. Statistical Analysis

The Pearson’s correlation coefficients were calculated in order to evaluate any significant correlations among the ARGs, bacterial community, and environmental parameters using R (version 3.3.1; stats package,). Redundancy analysis (RDA) was conducted using the vegan package in R. Network analysis was performed based on the Spearman’s correlation coefficients by Cytoscope 3.4.0 [39].

## 3. Results

### 3.1. Distributions of ARGs and the intI1 Gene 

**Figure 2:**
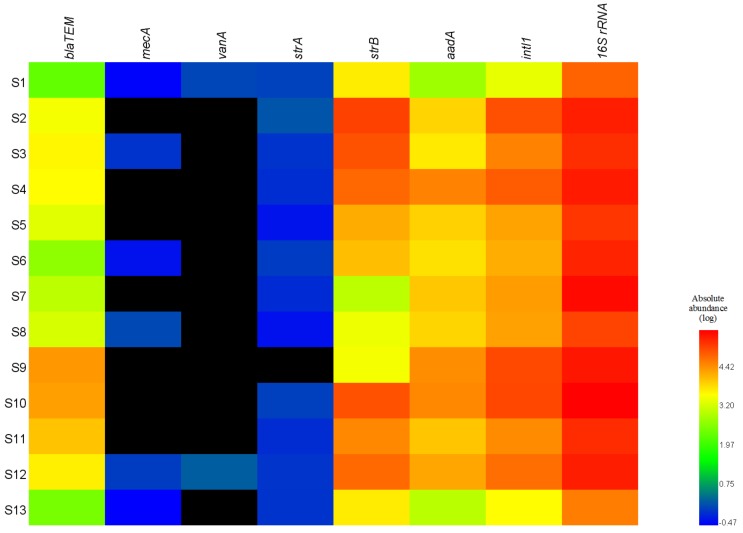
Heatmap showing the absolute abundances of antibiotic resistance genes (ARGs), *intI1*, and 16S rRNA genes in water samples collected from the Weihe River. Black denotes the absence of gene.

Figure 2 summarizes the absolute abundances of these genes detected at all the sampling sites. The total copy numbers of bacterial 16S rRNA genes in the surface water samples were quantified by normalization and they ranged from 4.38 × 10^4^ to 4.32 × 10^5^ copies/mL. Higher abundances of the *bla*_TEM_, *strB*, *aadA*, and *intI1* genes (10^3^–10^5^ copies/mL) was identified in the surface water samples compared with the relatively low abundances of the *strA*, *mecA*, and *vanA* genes (0–1.94 copies/mL) (Figure 2), thereby suggesting high-level contamination with β-lactam and streptomycin resistance genes. Spatially, the absolute abundances of *bla*_TEM_ gene were 122 and 102 times higher in the S9 (2.76 × 10^4^ copies/mL) and S10 (2.32 × 10^4^ copies/mL) samples than the S1 sample (*p* < 0.01, by Student’s *t*-test). Among the six ARGs detected, the *strB* gene was most abundant (1.32 × 10^5^ copies/mL) in the S2 sample (*p* < 0.01, by Student’s *t*-test), followed by the S10, S3, and S4 samples. The absolute abundances of the *aadA* gene were also significantly higher in the S4, S9, and S10 samples (3.94 × 10^4^, 3.31 × 10^4^, and 3.64 × 10^4^ copies/mL, respectively) compared with the S1 sample (*p* < 0.01, by Student’s *t*-test). The *intI1* gene was detected in all of the water samples (2.68 × 10^3^–1.20 × 10^5^ copies/mL) (Figure 2).

Figure S1 shows the distributions of the total relative abundances of ARGs and the *intI1* gene in the Weihe River, where the absolute abundances were normalized against the bacterial 16S rRNA gene copy number in the same sample in order to avoid underestimating the abundances due to the presence of eukaryotic DNA (i.e., protozoa, fungi, and algae). Spatially, the total relative abundances of the ARGs and the *intI1* gene were higher in the samples from Baoji urban district (S2, S3, and S4) than those from the other sites. The highest relative abundances of *strB* and *intI1* were found in S2 and S3, whereas the lowest were detected in S7. However, the total relative abundances of ARGs and *intI1* were relatively high at the sites ranging from S9 to S12 (Supplementary Materials Figure S1).

### 3.2. Microbial Diversity and Community Composition

In this study, after filtering the low-quality reads and trimming the chimeras, we obtained 20,520–206,956 high-quality tags from the 13 water samples using MiSeq sequencing (Supplementary Materials Table S3). According to the sequencing results, the numbers of OTUs ranged from 974 to 1849 in the 13 samples. Further analysis showed that 1083 shared OTUs collected at all sites from Baoji to Weinan, whereas 219, 276, and 278 OTUs were found only in Baoji, Xianyang and Xi’an, and Weinan, respectively (Supplementary Materials Figure S2), thereby suggesting that many of the same species were present in all 13 samples, but some unique species were also found in particular regions of the Weihe River.

**Figure 3:**
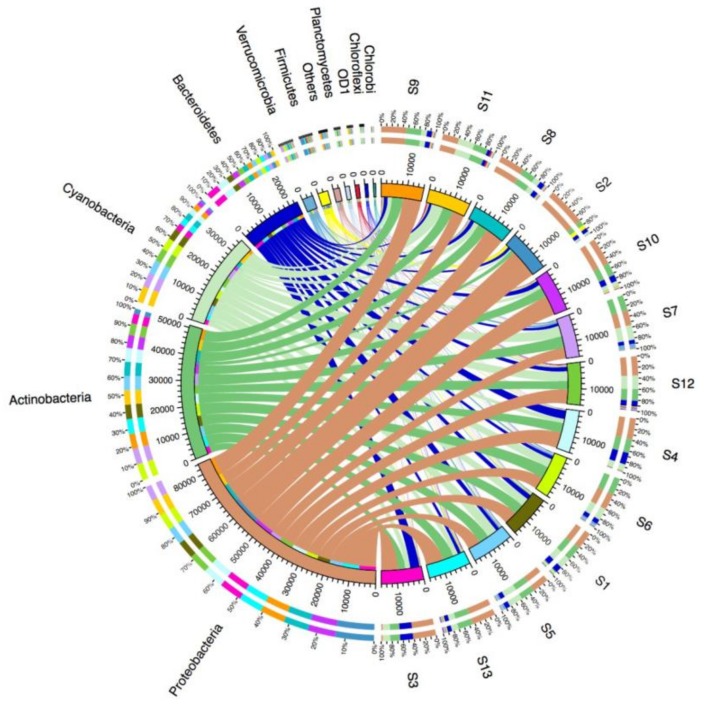
Distribution of phyla in the 13 water samples according to the taxonomic annotations obtained using the Greengenes database and the Ribosomal Database Project (RDP) classifier.

**Figure 4:**
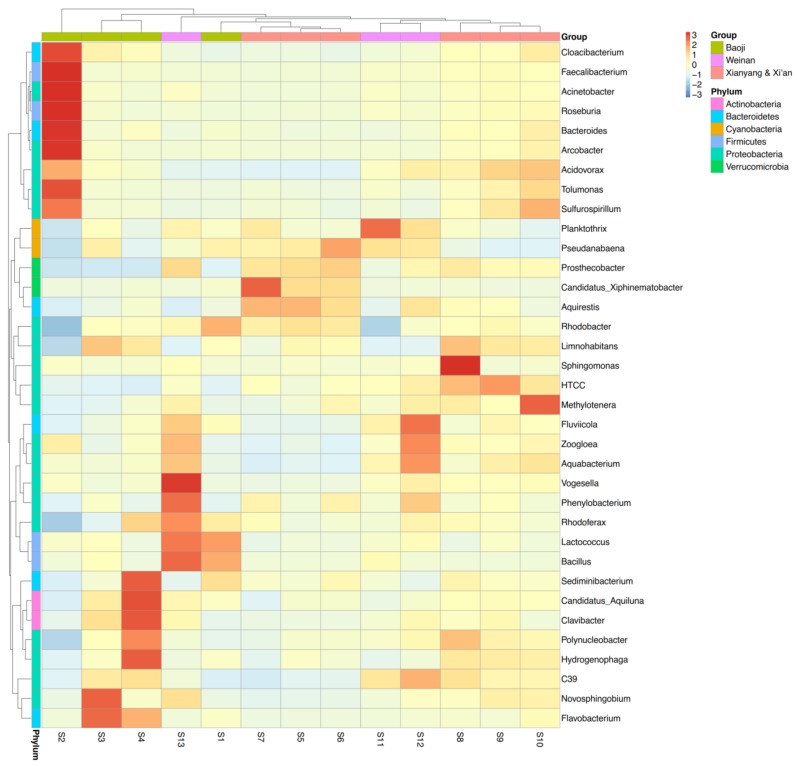
Heatmap showing the relative abundances of the 35 most abundant genera in the 13 water samples.

All of the effective sequences from each sample were further assigned to their corresponding taxonomic levels (from phylum to genus). The phylum distributions were visualized using Circos. As shown in Figure 3, Proteobacteria was the most abundant phylum in the 13 samples, where it accounted for 27.4–71.4% of the total effective sequences in different water samples. The other dominant phyla comprised Actinobacteria (9.3–30.6%), Cyanobacteria (3.7–29.5%), Bacteroidetes (6.4–23.7%), Firmicutes (0.56–7.1%), and Verrucomicrobia (0.25–5.5%). Spatially, Proteobacteria was clearly the most abundant phylum in S2, whereas Cyanobacteria was the least abundant. It should be noted that the phylum Firmicutes was more abundant at S2 than the other samples. The results obtained at the class, order and family levels showed that Epsilonproteobacteria, Campylobacterales, and Campylobacteraceae were the most abundant in the S2 bacterial community, respectively (Figure S3). Furthermore, we selected the top 35 dominant genera in the 13 samples and used them to prepare a heatmap (Figure 4). We found that *Cloacibacterium*, *Faecalibacterium*, *Acinetobacter*, *Roseburia*, *Bacteroides*, *Arcobacter*, *Acidovorax*, *Tolumonas*, and *Sulfurospirillum* (in the phyla Proteobacteria, Firmicutes and Bacteroidetes) were the dominant genera in S2. In particular the genera *Arcobacter* and *Acinetobacter* from the phylum Proteobacteria were the two most abundant genera in the S2 sample, where they accounted for 42.5% and 6.7% of the total effective sequences, respectively. These results indicate that the bacterial composition in S2 differed significantly from those in the other samples.

### 3.3. Relationships between ARGs, Bacterial Communities, and Environmental Factors

**Figure 5:**
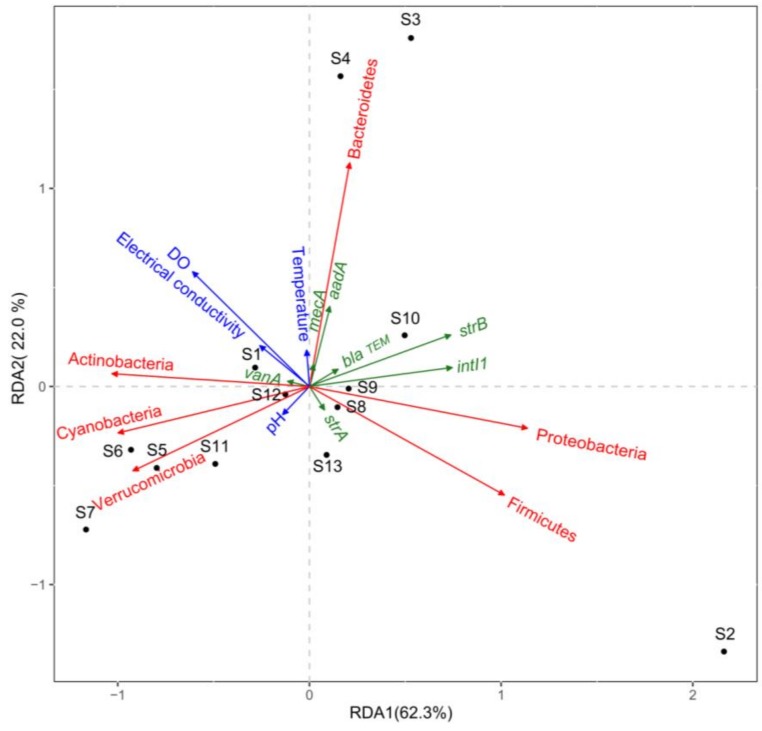
Redundancy analysis of the relationships between environmental factors (blue arrows), main bacterial phyla (red arrows), and ARGs and *intI1* genes (green arrows).

We employed RDA to investigate the relationships between the ARGs, bacterial communities, and environmental factors (electrical conductivity, temperature, pH and dissolved oxygen) in the water samples (Supplementary Materials Table S4). RDA analysis showed that the bacterial community and selected environmental factors explained 84.3% of the total variance in the ARGs (Figure 5). Clearly, the ARG profile for the S2 sample differed significantly from those of the other samples. In addition, the positive correlations between the phyla Proteobacteria and Firmicutes with the *strB* and *intI1* genes demonstrate that these phyla were the major factors responsible for driving the ARG distribution in S2 (Supplementary Materials Table S5). Similar to the microbial community compositions (Figure 4), the ARG profiles were clustered in S3 and S4, and they were positively associated with the phylum Bacteroidetes (Figure 5). According to the RDA results, the ARG profiles varied little between some of the water samples. For example, S1 was clustered with S8–S10 and S12–S13, whereas S11 was clustered with S5–S7. 

### 3.4. Network Analysis Based on the intI1 Gene, ARGs, and Bacterial Taxa

**Figure 6:**
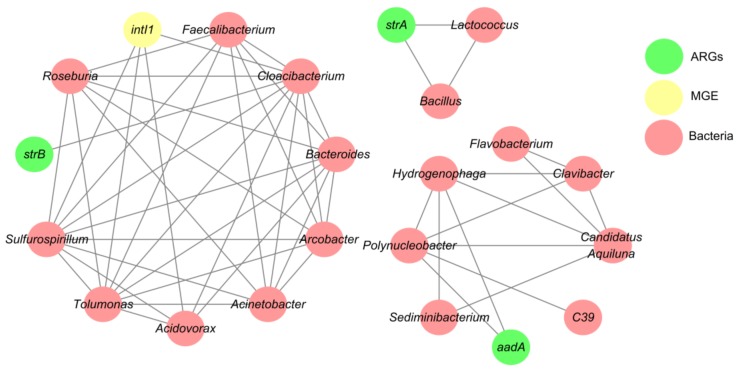
Network analysis showing the co-occurrence patterns based on the *intI1* gene, ARGs, and bacterial taxa in the 13 water samples.

The potential host bacteria for ARGs were identified using network analysis (Figure 6), which identified eight bacterial genera as possible hosts of the *intI1* gene and three ARGs (*strA*, *strB*, and *aadA*). Network analysis showed that 62.5% of the potential hosts for *intI1* and ARGs were Proteobacteria. *Lactococcus* and *Bacillus* were possible hosts of the streptomycin resistance gene *strA*, whereas *Cloacibacterium* could host the streptomycin resistance gene *strB*. In addition, the streptomycin resistance gene *aadA* was carried mainly by *Hydrogenophaga* and *Polynucleobacter*. The significant positive correlations between the *intI1* gene and the genera *Acidovorax*, *Cloacibacterium*, *Sulfurospirillum*, and *Tolumonus* suggest that these genera could have been hosts of the *intI1* gene. However, compared with the genes mentioned above, the *intI1* gene was carried by more diverse genera, thereby indicating that the *intI1* gene had a broader host range. 

## 4. Discussion

### 4.1. Prevalence of the intI1 Gene and ARGs in Urban Districts

In this study, we detected two β-lactam resistance genes (*bla*_TEM_ and *mecA*), one vancomycin resistance gene (v*anA*), three streptomycin resistance genes (*strA*, *strB*, and *aadA*), one integron-integrase gene (*intI1*), and the 16S rRNA gene in water samples from the Weihe River, Shaanxi, China using ddPCR. The *intI1* gene was detected in all of the water samples, which is consistent with the high levels found previously in urban rivers in Beijing and the Haihe River [14,40]. It has been reported that the abundance of the *intI1* gene is correlated with the levels of the *bla*_TEM_ and *aadA* genes [10,41], which was supported by our results (Supplementary Materials Table S6). Thus, the dissemination of the *bla*_TEM_ and *aadA* genes may be facilitated by the *intI1* gene, and HGT might explain the variations in the β-lactam and streptomycin resistance genes in the Weihe River. Furthermore, the *intI1* gene had a broader host range in Weihe River (Figure 6), thereby suggesting that HGT between bacterial hosts is a key factor responsible for the variations in ARGs in the Weihe River. Similar results were obtained in previous studies, which showed that class I integrons play important roles in the dissemination of ARGs in human-impacted aquatic environments [42]. 

In general, the *bla*_TEM_ gene is one of the most common antimicrobial resistance genes transferred by plasmids. The *bla*_TEM_ gene confers resistance to penicillins, cephalosporins and carbapenems, which account for approximately two-thirds by weight of the antibiotics consumed by humans [31,43]. The *strA*, *strB* and *aadA* genes are associated with resistance to streptomycin, which is one of the first-line antibiotics used for tuberculosis treatment. Streptomycin is still utilized widely in China. In addition, it has been used as an important agricultural bactericide since the late 1950s in both animal husbandry and plant disease control [44]. Therefore, agricultural pollution and discharges from fisheries may be the main sources of the high levels of the *strB* and *aadA* genes in the Weihe River. It should be noted that the absolute and relative abundances of the *strB* gene were significantly higher in our samples compared with those of *strA* (*p* < 0.01) (Figure 2), possibly because the abundances of the bacteria associated with the *strB* gene were higher in the Weihe River. This finding agrees with those obtained in a recent study, which showed that most streptomycin-resistant bacteria harbored the *strB* gene but not the *strA* gene [45].

### 4.2. Bacterial Community Diversity

Many previous studies have shown that Proteobacteria, Actinobacteria and Bacteroidetes are the most abundant bacterial phyla found in the communities in river water samples [46,47,48]. Similarly, we found that Proteobacteria, Actinobacteria, Cyanobacteria and Bacteroidetes were the most abundant phyla in the Weihe River using culture-independent methods based on 16S rRNA gene sequencing (Supplementary Materials Figure S3). The high-throughput sequencing results indicated that the bacterial composition of S2 differed significantly from those of the other samples, where Epsilonproteobacteria, Campylobacterales, Campylobacteraceae, and the genera *Arcobacter* and *Acinetobacter,* dominated the bacterial community. A previous study demonstrated that Campylobacteraceae (Epsilonproteobacteria) was the most abundant bacterial family in both treated and untreated wastewaters [49]. According to Li et al. [50], Epsilonproteobacteria might be specifically associated with antibiotic-containing aquatic environments. In addition, the genus *Arcobacter* includes both animal and human pathogens, which are found mainly in manure derived from animal livestock [51,52]. Several *Arcobacter* species in the phylum Epsilonproteobacteria are related to human infections and they often exhibit multi-drug resistance [53]. A previous study showed that *Acinetobacter* spp. are nosocomial pathogens with a higher frequency in wastewater than river water, thereby suggesting that the discharge of effluent wastewater can change the distribution of *Acinetobacter* spp. in the receiving water [54]. Thus, animal and human wastewaters along the Weihe River were probably the most important sources of the genera *Arcobacter* and *Acinetobacter*. The highest relative abundances of the *intI1* gene and ARGs were also detected in S2 (Supplementary Materials Figure S1), which was located near large-scale livestock and poultry farms, which indicates that anthropogenic activities may have caused changes in the bacterial community and increased the abundances of ARGs.

### 4.3. Relationships between ARGs, Bacterial Communities, and Environmental Factors 

The emergence and proliferation of ARGs are closely associated with the composition of the microbial community and environmental factors [55,56]. In the present study, RDA analysis showed that the bacterial community and selected environmental factors explained 84.3% of the total variance in the ARGs (Figure 5), but no significant correlations were found between the ARGs and environmental factors (Supplementary Materials Table S7). Therefore, compared with the environmental factors, the bacterial communities probably made the largest contribution to the dynamic changes in ARGs, as also concluded in a previous study [57] where the bacterial community was the major factor responsible for driving the ARG distribution in aquatic environments.

In order to understand the detailed relationships between specific ARGs and bacteria, we identified the potential host bacteria for ARGs using network analysis. Some of our results were consistent with those obtained in previous studies of antibiotic resistance. For example, *Lactococcus* and *Hydrogenophaga* strains were previously shown to exhibit resistance to streptomycin [58,59], and the *strA* gene was detected at a high frequency in *Bacillus* strains isolated from the influent samples in wastewater treatment processes [59]. Network analysis is a reasonable and powerful tool for obtaining new insights into the distributions of ARGs and their possible hosts in complex environmental samples [60], and it should be noted that some of the suggested hosts shown in Figure 6 were determined for the first time in the present study. Therefore, additional research is required to isolate the bacterial hosts of ARGs in various environments [39] and the relationships identified by network analysis should also be validated using other approaches.

## 5. Conclusions

β-lactam (*bla*_TEM_) and streptomycin (*strB* and *aadA*) resistance genes were highly prevalent in the Weihe River, where the highest relative abundances of the *intI1* gene and ARGs were detected in Baoji urban district. The bacterial community exhibited spatial variation along the Weihe River basin, and the bacterial composition of the samples from Baoji urban district differed significantly from those of the other samples. Our results also suggest that the bacterial communities and HGT explained most of the variations in ARGs. 

## Supplementary Materials

The following are available online at http://www.mdpi.com/1660-4601/15/4/708/s1. Figure S1: Total relative abundances of ARGs and *intI1* in water samples collected from the Weihe River; Figure S2: Venn diagrams showing the shared and distinct operational taxonomic units (OTUs) in different sites; Figure S3: Distributions of the relative abundances of the 10 most abundant bacteria in the 13 samples at different levels: (a) phylum level; (b) class level; (c) order level; and (d) family level; Table S1: Descriptions of the sampling sites; Table S2: Primers and probes for the detection of clinically relevant antibiotic genes in this study; Table S3: Raw and clean tags, OTUs, Good’s coverage, and Shannon, Chao1, ACE, and Simpson’s indices for the 13 water samples; Table S4: Environmental factors used for redundancy analysis; Table S5: Pearson’s correlation coefficients between genes and the main bacterial phyla. * Significantly different at *p* < 0.05. ** Significantly different at *p* < 0.01; Table S6: Pearson’s correlation coefficients between the relative abundances of ARGs and the *intI1* gene. * Significantly different at *p* < 0.05; Table S7: Pearson’s correlation coefficients between genes and environmental factors. * Significantly different at *p* < 0.05.
