# Supplementary material for: Abundances of Clinically Relevant Antibiotic Resistance Genes and Bacterial Community Diversity in the Weihe River, China
Source: Int J Environ Res Public Health. 2018 Apr 10;15(4):708. doi: 10.3390/ijerph15040708 (PMC5923750; doi:10.3390/ijerph15040708)
Supplement: Supplementary file 1 [file ijerph-15-00708-s001.pdf]

International Journal of
Environmental Research and Public Health

MDPI

Supplementary Materials: Abundances of Clinically Relevant Antibiotic Resistance Genes and Bacterial Community Diversity in the Weihe River, China

Xiaojuan Wang [1], Jie Gu [1,*], Hua Gao[1], Xun Qian[1] and Haichao Li [1]

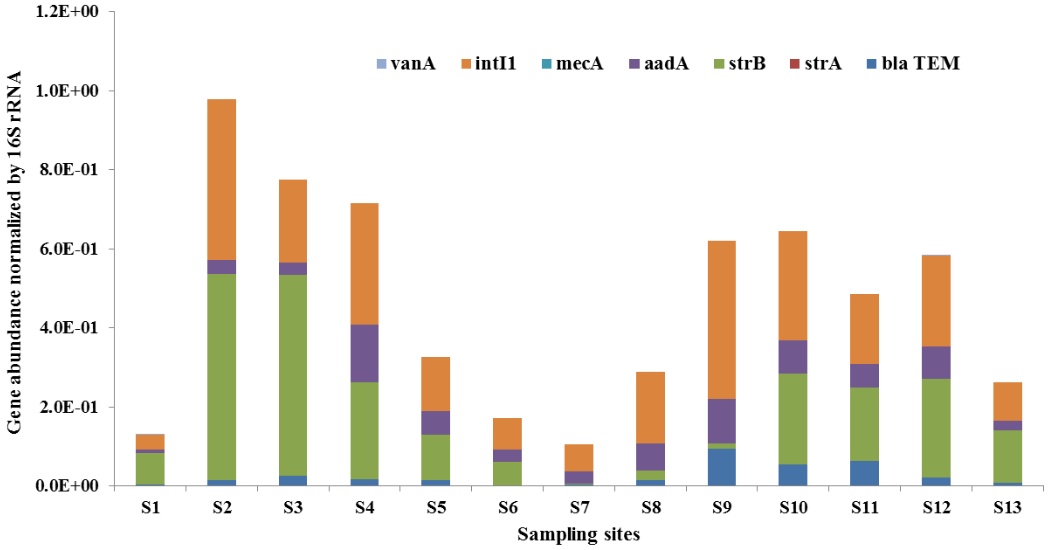

Figure S1. Total relative abundances of ARGs and *intI1* in water samples collected from the Weihe River.

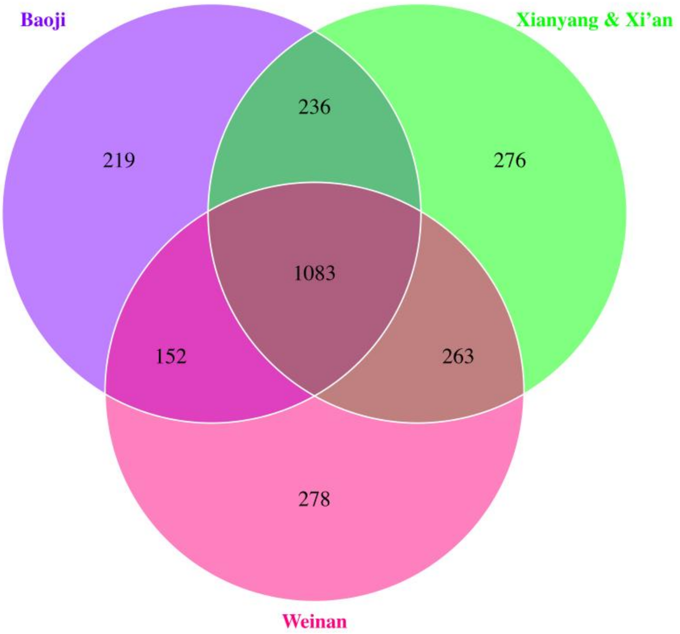

Figure S2. Venn diagrams showing the shared and distinct operational taxonomic units (OTUs) in different sites.

(a)

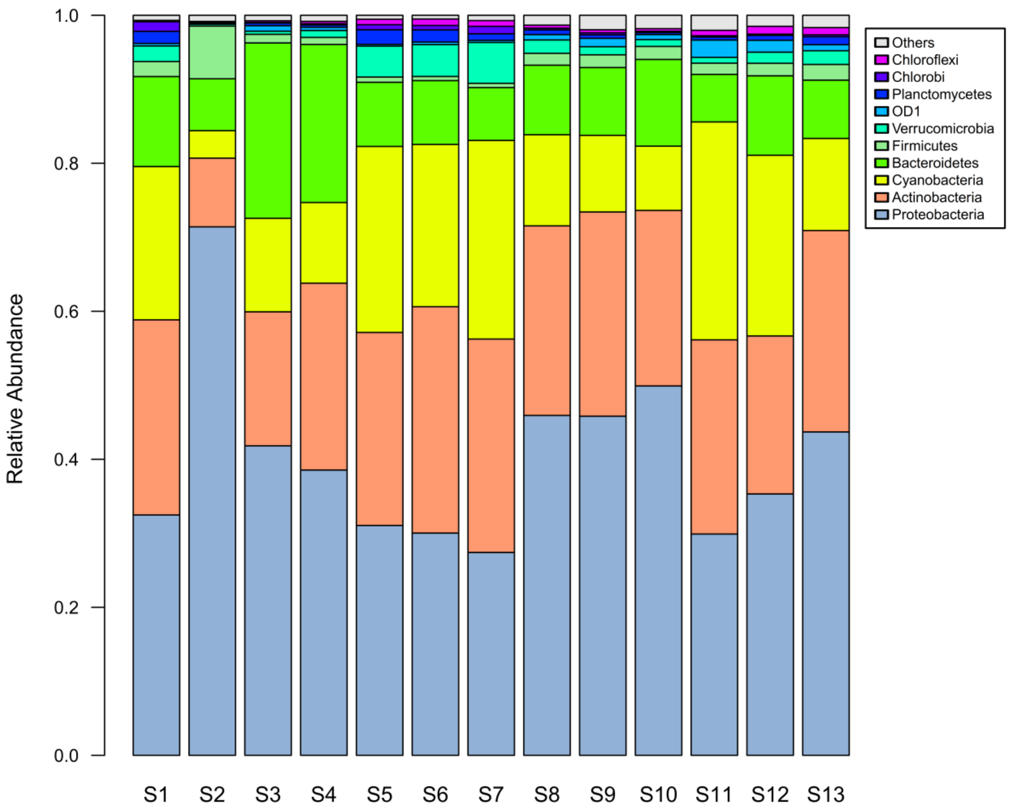

Relative Abundance
1.0
0.8
0.6
0.4
0.2
0.0
S1 S2 S3 S4 S5 S6 S7 S8 S9 S10 S11 S12 S13
Others
Chloroflexi
Chlorobi
Planctomycetes
OD1
Verrucomicrobia
Firmicutes
Bacteroidetes
Cyanobacteria
Actinobacteria
Proteobacteria

(b)

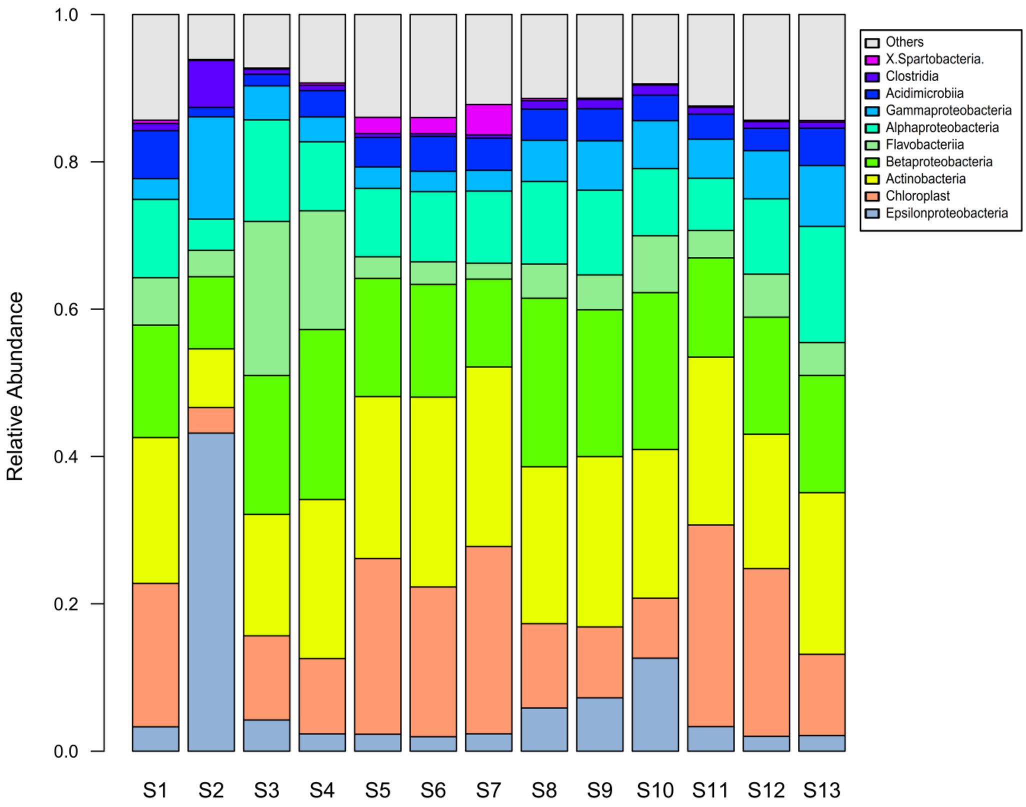

Relative Abundance
1.0
0.8
0.6
0.4
0.2
0.0
S1 S2 S3 S4 S5 S6 S7 S8 S9 S10 S11 S12 S13
Others
X.Spartobacteria.
Clostridia
Acidimicrobiia
Gammaproteobacteria
Alphaproteobacteria
Flavobacteriia
Betaproteobacteria
Actinobacteria
Chloroplast
Epsilonproteobacteria

(c)

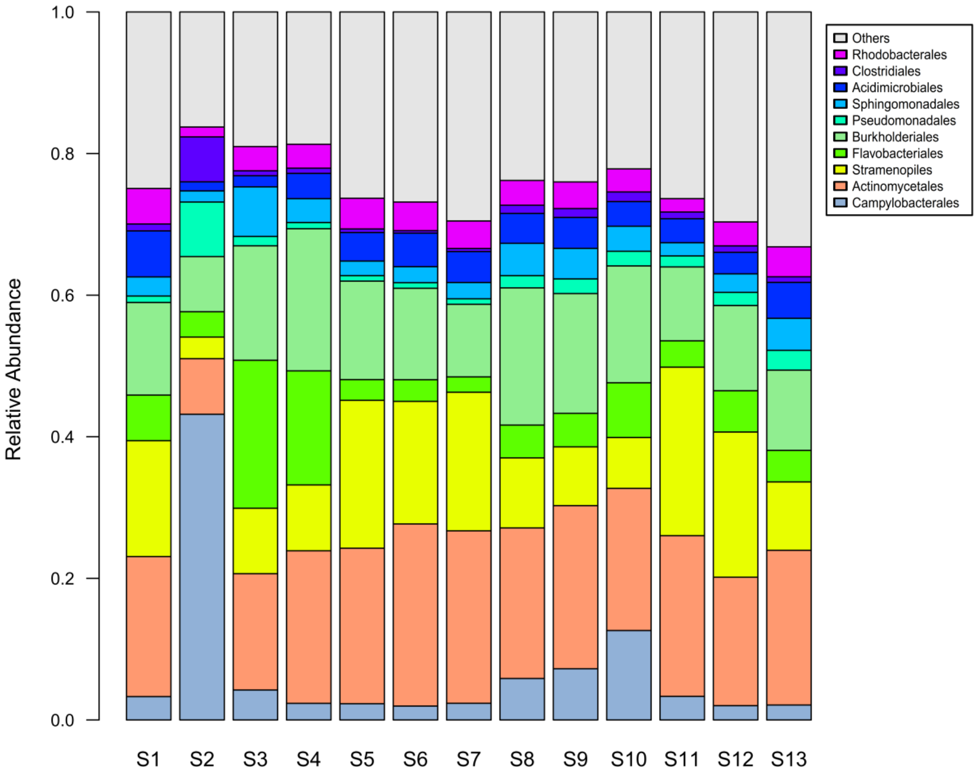

(d)

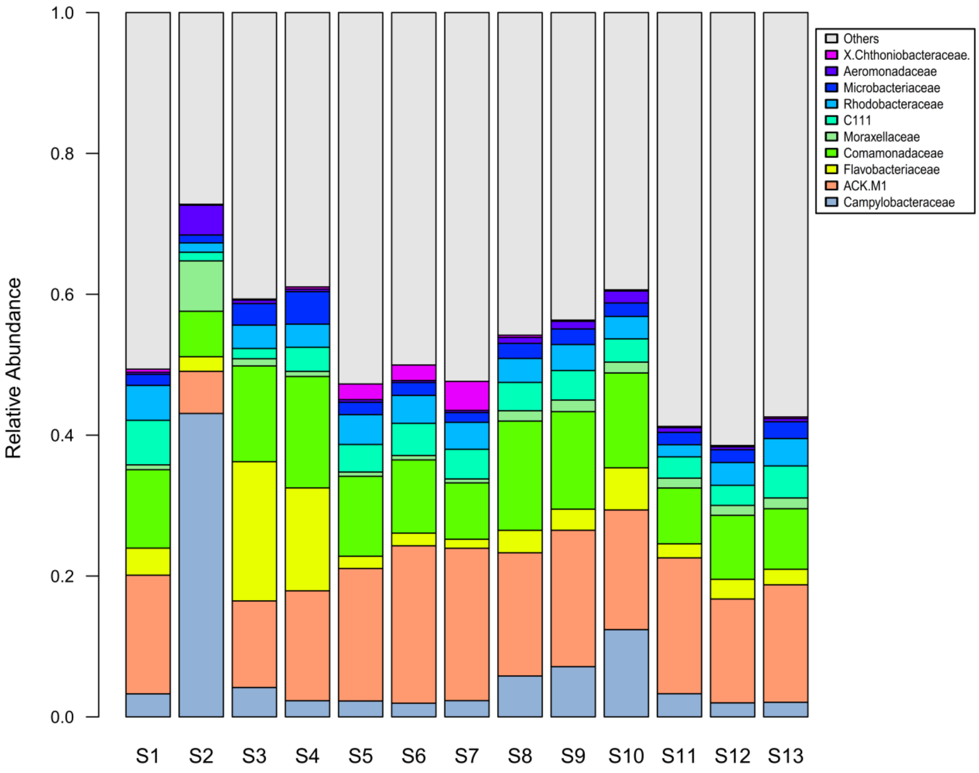

Figure S3. Distributions of the relative abundances of the 10 most abundant bacteria in the 13 samples at different levels: (**a**) phylum level; (**b**) class level; (**c**) order level; and (**d**) family level.

Table S1. Descriptions of the sampling sites.

City	Sample ID	Site	Urban/Rural	Location
Baoji	S1	Linjia Village	Rural	107.05°E 34.36°N
	S2	Wolongsi Bridge	Urban	107.27°E 34.34°N
	S3	Guozhen Bridge	Urban	107.39°E 34.33°N
	S4	Changxing Bridge	Rural	107.83°E 34.21°N
Xianyang and Xi'an	S5	Xingping	Rural	108.51°E 34.23°N
	S6	Nanying	Rural	108.65°E 34.28°N
	S7	Xianyang Railway Bridge	Rural	108.81°E 34.37°N
	S8	Tianjiangrendu	Urban	108.97°E 34.40°N
	S9	Gengzhen Bridge	Urban	109.11°E 34.45°N
	S10	Xinfeng Bridge	Urban	109.26°E 34.47°N
Weinan	S11	Shawangdu	Urban	109.50°E 34.53°N
	S12	Shuyuan	Urban	109.60°E 34.52°N
	S13	Tongguan Suspension Bridge	Rural	110.24°E 34.62°N

Table S2. Primers and probes for the detection of clinically relevant antibiotic genes in this study.

Class	Target gene	Primer sequence (5′→3′)	Probe sequence	Amplicon size (bp)	Reference
β-lactam	*bla*$_{TEM}$	F-CACTATTCTCAGAATGACTTGGT<br>R-TGCATAATTCTCTTACTGTCATG	FAM-CCAGTCACAGAAAAGCATCTTACGG-BHQ1	85	[31]
	mecA	F-CGCAACGTTCAATTTAATTTTGTTAA<br>R-TGGTCTTTCTGCATTCCTGGA	VIC-AATGACGCTATGATCCCAATCTAACTTCCACA-BHQ1	92	[32]
vancomycin	*vanA*	F-CTGTGAGGTCGGTTGTGCG<br>R-TTTGGTCCACCTCGCCA	FAM-CAACTAACGCGGCACTGTTTCCCAAT-BHQ1	64	[33]
streptomycin	*strA*	F-TCAATCCCGACTTCTTACCG<br>R-CACCATGGCAAACAACCATA	FAM-TGCTCGACCAAGAGCGGC-BHQ1	126	[34]
	strB	F-ATCGCTTTGCAGCTTTGTTT<br>R-ATGATGCAGATCGCCATGTA	VIC-ATGCCTCGGAACTGCGT-BHQ1	143	
	aadA	F-CAGCGCAATGACATTCTTGC<br>R-GTCGGCAGCGACA(C/T)CCTTCG	FAM-TGGTAGGTCCAGCGGCGGAG-BHQ1	295	
intI1		F-GCCTTGATGTTACCCGAGAG<br>R-GATCGGTCGAATGCGTGT	VIC-ATTCCTGGCCGTGGTTCTGGGTTTT-BHQ1	196	[35]
16S rRNA		F-TCCTACGGGAGGCAGCAGT<br>R-GGACTACCAGGGTATCTAATCCTGTT	VIC-CGTATTACCGCGGCTGCTGGCAC-BHQ1	466	[34]

Table S3. Raw and clean tags, OTUs, Good's coverage, and Shannon, Chao1, ACE, and Simpson's indices for the 13 water samples.

Sample ID	Raw tags	Effective tags	OTU number	Good's coverage	Shannon	Chao 1	ACE	Simpson
S1	147375	75725	1471	0.978	6.747	1316.694	1395.271	0.961
S2	412139	206956	1849	0.98	5.488	1189.152	1244.642	0.869
S3	90719	42527	1148	0.982	6.489	1067.38	1127.118	0.967
S4	45508	20520	974	0.97	6.73	2835.082	1746.054	0.97
S5	103522	53191	1270	0.98	6.37	1242.435	1282.704	0.944
S6	215539	107237	1554	0.979	6.476	1290.15	1303.588	0.953
S7	67992	33923	996	0.981	6.218	1179.19	1180.159	0.944
S8	141226	65258	1617	0.975	6.995	1453.173	1559.622	0.974
S9	52020	23979	1140	0.985	7.041	1092.96	1132.75	0.976
S10	63319	29698	1190	0.981	6.864	1141.638	1275.748	0.973
S11	60770	32636	1424	0.976	6.616	1494.455	1553.858	0.933
S12	48273	24085	1286	0.982	6.921	1234.444	1314.747	0.951
S13	93267	46152	1584	0.977	7.619	1498.083	1568.162	0.98

Table S4. Environmental factors used for redundancy analysis.

Sample ID	Electrical conductivity (μs/cm)	Temperature (°C)	pH	Dissolved oxygen (mg/L)
S1	744	18.2	7.62	10.42
S2	755	20.0	8.57	3.56
S3	925	21.5	8.69	8.82
S4	829	19.7	8.10	11.62
S5	880	18.4	8.86	9.26
S6	891	19.7	8.70	9.63
S7	874	20.3	8.91	9.40
S8	808	14.8	8.18	10.00
S9	656	13.2	7.88	8.45
S10	975	12.2	7.97	8.06
S11	874	13.8	7.85	6.58
S12	950	14.8	7.96	7.67
S13	885	13.2	7.98	7.78

Table S5. Pearson's correlation coefficients between genes and the main bacterial phyla * Significantly different at $p < 0.05$. **Significantly different at $p < 0.01$.

	bla TEM	*strA*	*strB*	*aadA*	*mecA*	*intI1*	*vanA*
Proteobacteria	0.154	0.04	0.575*	0.089	0.024	0.729**	−0.238
Actinobacteria	0.049	0.025	−0.891**	0.08	0.08	−0.59*	0.038
Cyanobacteria	−0.15	−0.073	−0.439	−0.26	−0.119	−0.670*	0.247
Bacteroidetes	−0.070	−0.093	0.469	0.302	0.064	0.167	0.050
Verrucomicrobia	−0.464	−0.063	−0.631*	−0.341	−0.091	−0.676*	−0.013
Firmicutes	−0.023	0.214	0.574*	−0.181	−0.064	0.534	0.027

Table S6. Pearson's correlation coefficients between the relative abundances of ARGs and the *intI1* gene. * Significantly different at $p < 0.05$.

	bla TEM	*mecA*	*vanA*	*strA*	*strB*	*aadA*	*intI1*
*bla*TEM	1.000						
mecA	−0.370	1.000					
vanA	−0.249	0.254	1.000				
strA	−0.443	0.534	0.480	1.000			
strB	−0.051	−0.144	−0.122	−0.008	1.000		
aadA	0.515	−0.318	−0.296	−0.573*	−0.051	1.000	
intI1	0.583*	−0.381	−0.354	−0.468	0.483	0.640*	1.000

Table S7. Pearson's correlation coefficients between genes and environmental factors. * Significantly different at $p < 0.05$.

	bla TEM	*strA*	*strB*	*aadA*	*mecA*	*intI1*	*vanA*
pH	−0.425	−0.348	0.164	−0.290	−0.238	−0.141	−0.497
Dissolved oxygen	−0.235	−0.041	−0.536	0.246	0.216	−0.44	0.210
Temperature	−0.599*	−0.084	0.342	−0.300	−0.188	−0.161	0.032
Electrical conductivity	−0.261	−0.063	0.224	−0.121	0.038	−0.335	−0.197

© 2018 by the authors. Submitted for possible open access publication under the terms and conditions of the Creative Commons Attribution (CC BY) license (http://creativecommons.org/licenses/by/4.0/).
